# Global gene expression in pseudomyxoma peritonei, with parallel development of two immortalized cell lines

**DOI:** 10.18632/oncotarget.3198

**Published:** 2015-04-13

**Authors:** Darren L. Roberts, Sarah T. O'Dwyer, Peter L. Stern, Andrew G. Renehan

**Affiliations:** ^1^ Immunology Group, Paterson Institute for Cancer Research, The University of Manchester, Manchester, M20 4BX, UK; ^2^ Institute of Cancer Sciences, The University of Manchester, Manchester Academic Health Science Centre, The Christie NHS Foundation Trust, Manchester M20 4BX, UK; ^3^ Peritoneal Tumour Service, Department of Surgery, The Christie NHS Foundation Trust, Manchester, M20 4BX, UK

**Keywords:** pseudomyxoma peritonei, cell line, characterization, exon array

## Abstract

Pseudomyxoma peritonei (PMP) is a rare tumor of appendiceal origin. Treatment is major cytoreductive surgery but morbidity is high. PMP is considered chemo-resistant; its molecular biology is understudied; and presently, there is no platform for pre-clinical drug testing. Here, we performed exon array analysis from laser micro-dissected PMP tissue and normal colonic epithelia. The array analysis identified 27 up-regulated and 34 down-regulated genes: candidate up-regulated genes included SLC16A4, DSC3, Aldolase B, EPHX4, and ARHGAP24; candidate down-regulated genes were MS4A12, TMIGD1 and Caspase-5. We confirmed differential expression of the candidate genes and their protein products using *in-situ* hybridization and immuno-histochemistry. In parallel, we established two primary PMP cell lines, N14A and N15A, and immortalized with an SV40 T-antigen lentiviral vector. We cross-checked for expression of the candidate genes (from the array analyses) using qPCR in the cell lines and demonstrated that the gene profiles were distinct from those of colorectal tumor libraries and commonly used colon cell lines. N14A and N15A were responsiveness to mitomycin and oxaliplatin. This study characterizes global gene expression in PMP, and the parallel development of the first immortalized PMP cell lines; fit for pre-clinical testing and PMP oncogene discovery.

## INTRODUCTION

Pseudomyxoma peritonei (PMP) is a rare epithelial neoplasm, of mainly appendiceal origin [1], which if untreated, is characterized by disseminated peritoneal mucinous tumor deposition and progressive accumulation of mucinous ascites [2]. Distant and nodal metastases are the exception. PMP has an estimated incidence of 1–2 per million in Western populations [3]. In nationally representative series [4, 5], 30–70% of patients at presentation are suitable to undergo loco-regional surgical treatment involving the combination of macroscopic tumour excision, described as cytoreductive surgery (CRS), and hyperthermic intraperitoneal chemotherapy (HIPEC)—often referred to as the “Sugarbaker procedure”, following his work in developing the techniques involved [6].

Due to the rarity of this tumor, biological understanding is limited. There have been a small number of reports on PMP that focused on: (i) selected pathways determining protein expression, for example, mucins [7–9]; epithelial-mesenchymal transition proteins [7, 8]; and CDX2 expression [10]; and (ii) on selected genetic mutations, for example, quantifying the proportion of samples with *K-RAS* [11, 12], *GNAS* [13], and *p53* mutations [14]—but no global characterisation approaches.

For chemotherapy options, PMP is generally considered to be resistant. During HIPEC administration, the commonest used agent is mitomycin C [15], though other agents including oxaliplatin and cisplatin, with and without concurrent systemic 5-fluorocuracil (a fluoropyrimidine), are also administered [6]. By the intra-peritoneal route, these agents are delivered in concentrations considerably higher than those used systemically. The rationale for their selection is based on empirical extrapolation from treatments of colorectal cancer. Despite the radicality of CRS and HIPEC, there is a recognized propensity for disease recurrence and progression. For the latter, and in patients deemed unsuitable for initial radical surgery, the natural history is characterized by high levels of morbidity (e.g. abdominal distension, discomfort, fistulation), and demise due to disease progression. We have previously reported a phase II trial in this setting, using systemically combined mitomycin and oral fluoropyrimidine-based chemotherapy, capecitabine (MCap), but with short-term stabilization of disease of a few months in only a third of patients [16]. Against this background, there is a clear need to improve the effectiveness of current chemotherapy regimens and/or develop new anti-PMP agents.

In this study, we address the two aforementioned needs in translational research for PMP. First, we performed exon array analysis from laser micro-dissected PMP tissue and comparative normal colonic epithelia; identified and confirmed differential expression of the candidate genes and their protein products in tissue. In parallel, we established two primary PMP cell lines. From our previously experiments [7], we learnt that primary PMP cell lines are slow-growing cells, with limited viability, and unfit for high-throughput experiments. Thus, here, we immortalized these cell lines with an SV40 T-antigen lentiviral vector, and cross-checked for differentially expressed genes, from the array analyses, using qPCR.

## RESULTS

**Figure 1:**
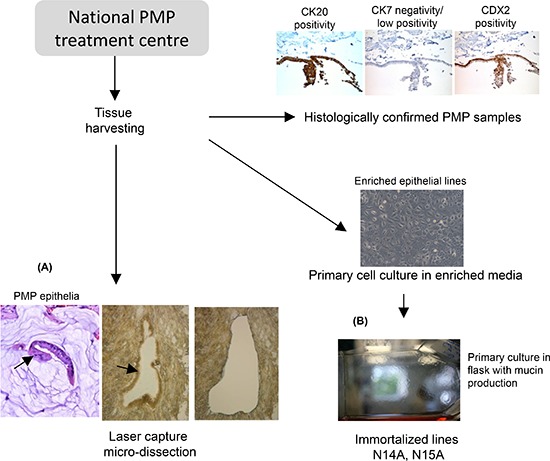
Overview flow diagram of the tissue harvest and cell line studies It is technically challenging to work with PMP epithelial tissue as it exists in small clusters in an ‘ocean’ of mucin (arrow in H&E section). **(A)** We developed laser capture micro-dissection methods to maximise epithelial yield from specimens that were confirmed histologically as PMP. In parallel, we generated primary PMP cell cultures from confirmed PMP tissue. **(B)** shows that the immortalized cells exhibit the characteristic “cobblestone” epithelial morphology cells, and produce a layer of mucin on the flask—equivalent to the clinical phenotype.

It is technically challenging to work with PMP epithelial tissue as it exists in small clusters in an ‘ocean’ of mucin. We developed laser capture micro-dissection methods to maximize epithelial yield from specimens that were confirmed histologically as PMP (Figure 1A).

### Gene microarray analysis

**Figure 2:**
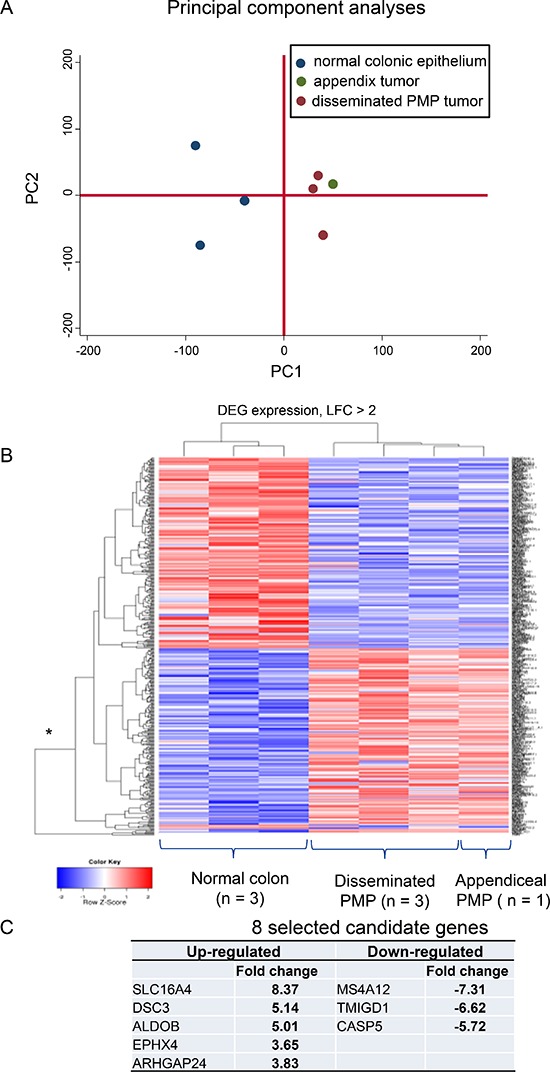
Exon array analysis identified 27 up-regulated and 34 down-regulated genes in PMP epithelial tissue (*p* < 0.05, adjusted for multiple testing) compared with normal colonic mucosa There was a high levels of homogeneity demonstrated by **(A)** the distinctive clustering between normal and neoplastic tissue in the principal component analysis and **(B)** the shallow heights of the DEG expression bars. **(C)** Eight candidate genes were selected and explored in greater detail. DEG: differentially expressed genes; LFC: log fold change.

We performed exon-array analysis comparing three disseminated (all omentum) plus one appendiceal PMP samples versus three samples of normal colonic mucosa. Initial PCA plots of the expressed genes demonstrated that the normal versus omental samples clustered to distinct populations at both the probeset and gene level (Figure 2A). These differences were not due to adipose tissue contamination of the omentum samples, as when the appendiceal PMP sample was added, it clustered with the omentum samples suggesting true differences between normal and diseased states. Overall, there was a high level of homogeneity (see Figure 1 Legend).

The differences in expression of the identified genes with greater than two fold changes were visualized using a heat-map (Figure 2B). For disease PMP tissue versus normal, 450 genes were identified as differentially expressed. The differential expressions were similar whether or not the appendiceal sample was included. After adjustment for multiple testing, 27 genes up-regulated in PMP were statistically significant with *p* values less than 0.05; thirty four genes were significantly down-regulated. These are listed, with descriptions of their main biological functions, in Supplementary Table S1. From these lists, we selected to explore in greater detail eight genes, based on (i) statistical significance; (ii) known biological function; and (iii) availability of probes and antibodies for validation (Figure 2C). The selected up-regulated genes were: SLC16A4, a proton-linked monocarboxylate transporter; DSC3 (desmocollin 3), a component of intercellular desmosome junctions; ALDOB, a fructose-1,6-bisphosphate aldolase; EPHX4, a hydrolase; and ARHGAP24, a Rho GTPase-activating protein involved in cell polarity, cell morphology and cytoskeletal organization. The commonly used PMP marker, MUC2, was increased by 1.8 log fold increase in the PMP samples compared with normal colonic mucosa expression, just outside our *a priori* 2-fold cut-off. The down-regulated genes were: MS4A12, a component of a multimeric receptor complex; TMIGD1, a transmembrane and immunoglobulin domain-containing protein-1; and Caspase-5, a mediator of apoptosis.

### Gene and protein product expression in PMP tissue

**Figure 3:**
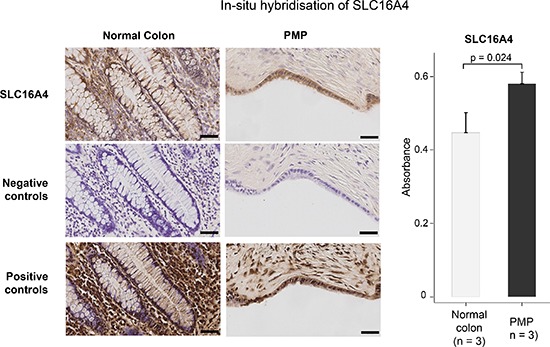
*In-situ* hybridization staining for gene expression of up-regulated gene, SLC16A4, in normal colon and disseminated PMP, with positive and negative control probes Scale bar represents 100 μm on low power images and 50 μm on high power images. Data in the graphs represent the mean ± SD for SLC16A4 expression by PMP versus normal colonic mucosa. Absorbance determined using the Definiens software. For the PMP tissue, a correction was made to account for the dilutional effect of mucin. *p* value derived from Student *t*-test.

To validate the above observed differences in gene expression, and in the absence of a suitable antibody, we performed *in-situ* hybridization (ISH) for the top up-regulated gene, SLC16A4, on PMP (*n* = 3) versus normal colonic mucosa (*n* = 3) samples (Figure 3). This confirmed our findings from the exon array analysis, that expression of SLC16A4 is elevated in PMP.

For immunohistochemical (IHC) staining, there was no difference in expression intensities between PMP and normal colonic epithelia for the clinically used tissue markers, CDX2, MUC2 and KRT20. However, the distribution of MUC2 is altered in PMP: MUC2 expression limited to goblet cells in normal intestinal tissue, but universally expressed in PMP cells, consistent with the notion that PMP is a goblet cell neoplastic process. Additionally, whereas KRT20 is limited to the colonic table in normal colonic epithelia, its expression is ubiquitous in PMP (Supplementary Figure S1).

**Figure 4:**
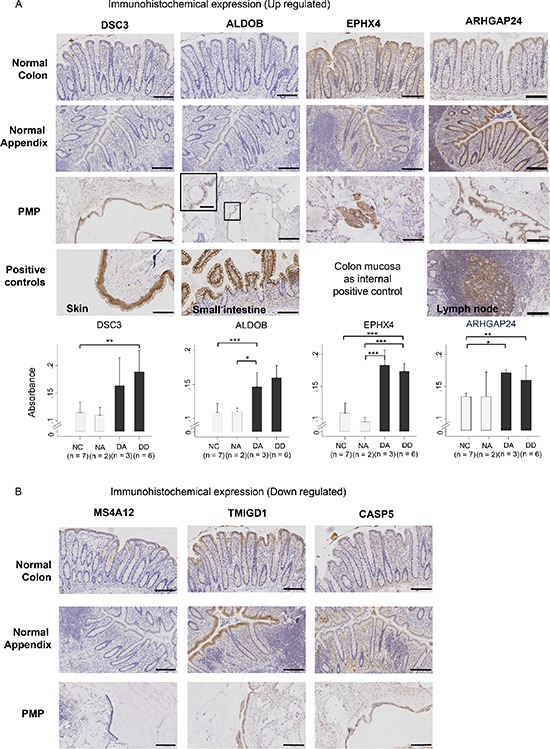
Immuno-histochemical staining for protein product expression of (A) up-regulated genes (DSC3, ALDOB, EPHX4, ARHGAP24) and (B) down-regulated genes (MS4A12, TMIGD1, CASP5) in normal colon (NC) and normal appendiceal (NA) mucosa, diseased appendix (DA), and disseminated disease (DD), with positive and negative controls Data in the graphs represent the mean ± SD, and differences tested by Student's *t*-test between comparative pairs from either diseased groups (DA, DD) versus normal epithelia groups (NC, NA) (*p < 0.01; **p < 0.001; ***p < 0.0001). Absorbance determined using the Definiens software. Scale bar represents 100 μm on low power images and 50 μm on high power images.

Image analysis of the staining for DSC3 (desmocollin 3), Aldolase B, EPHX4 and ARHGAP24 generally agreed with the exon array demonstrating significant increases in PMP tissue (3 diseased appendix; 6 disseminated disease) compared with either normal colonic (*n* = 7) or normal appendiceal (*n* = 2) epithelia (Figure 4A). IHC staining for the top down-regulated gene, MS4A12, revealed apical membrane staining of the colonocytes in normal colon samples, as previously described [17], and an absence of staining in the PMP samples consistent with the exon array results (Figure 4B). Comparative expressions for other markers (TMIGD1 and Caspase-5), which were down-regulated in the gene analysis, were equivocal on IHC due to the low levels of expression in the normal tissue and background staining of the mucin component of the PMP samples, and no formal statistics were performed.

### Establishment of two immortalized PMP cell lines

In parallel, we developed cell lines from samples from patients with histological confirmed PMP. Lines were established from multiple patients and multiple disease sites per patient resulting in approximately 50 primary cell lines. Of these, two sustainable cell lines underwent further characterization, N14A and N15A, both derived from primary appendiceal disease tissue, each from separate patients. Immortalized cells exhibit the characteristic “cobblestone” epithelial morphology cells, produce a layer of mucin on the flask (Figure 1B) (but do not stain for MUC2 due to the highly glycosylated nature of the mucin), and have a doubling time of approximately 48 h after clonal expansion. The short tandem repeats (STR) ‘finger printing’ profile of the cell lines was matched to genomic DNA isolated from FFPE samples from the same patients, and confirm identical sources. Additionally, the STR profiles did not match known cell line profiles from the ATCC (data available from authors on request).

### Cross-validation of differential gene expression in cell lines

**Figure 5:**
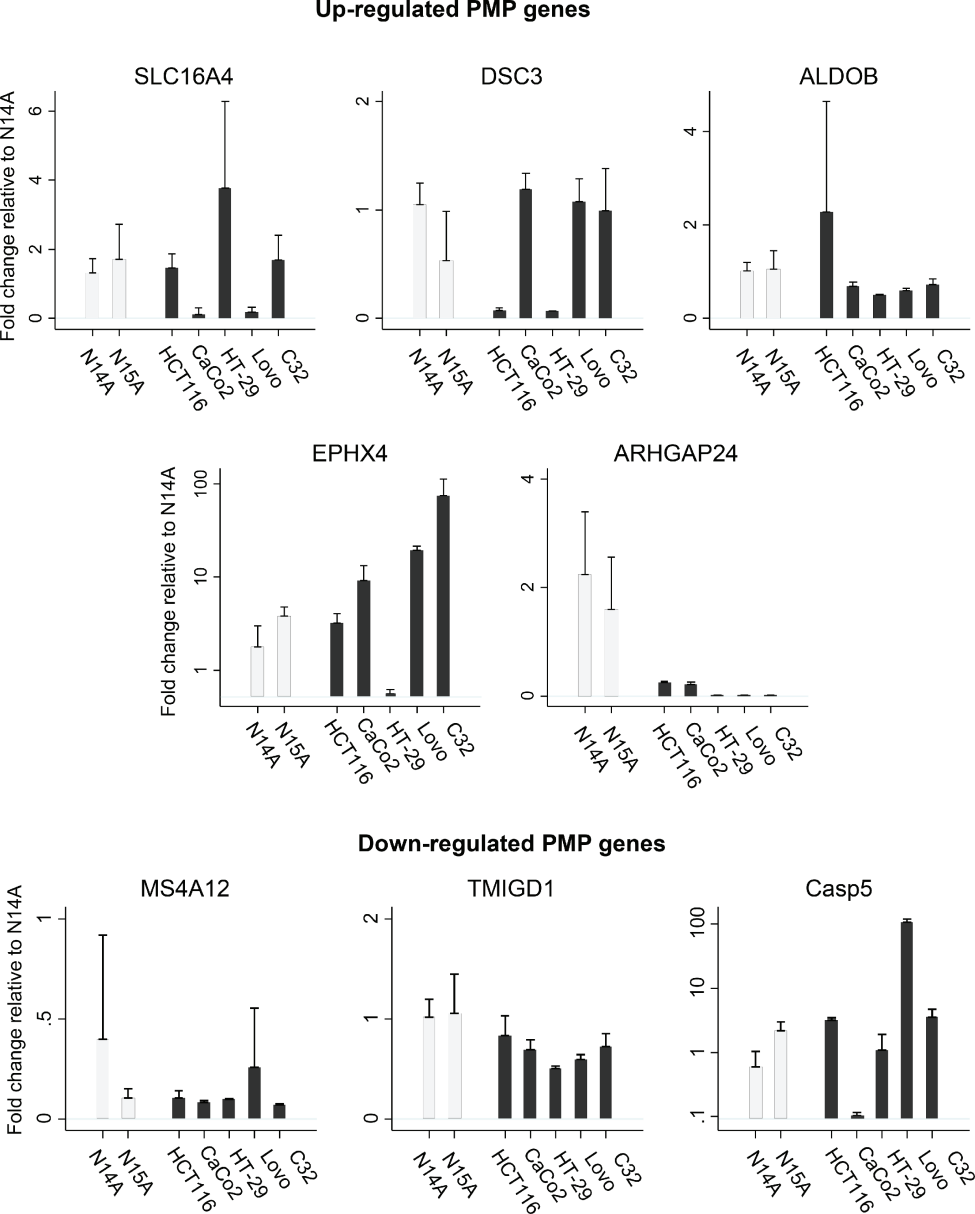
In order to validate the comparative analyses observed in the Oncomine database, we determined qPCR gene expression for candidate up-regulated and down-regulated PMP genes in N14A and N15A relative to 5 colon cancer cell lines In the absence of a widely available human normal intestinal lines, there is no natural comparator group. Nonetheless, the gene expression is relatively consistent for the two PMP cell lines, but differ considerable compared with colon cell lines for each candidate gene. For plots of up-regulated PMP genes, data have been standardised against ALDOB N14A; for plots of down-regulated PMP genes, data have been standardised against TMIGD1 N14A. Note different y-axis scales.

The PMP cell lines were screened by qPCR for levels of our candidate eight genes. In the absence of a widely available human normal intestinal lines, there is no natural comparator group. We thus compared with the same gene expressions (up-regulated transcripts: SLC16A4, DSC3, ALDOB, EPHX4, ARHGAP24; and down-regulated transcripts (MS4A12, TMIGD1, CASP5) in 5 colon cancer lines: HCT 116, Caco2, HT-29, Lovo, and C32. In general, gene expression of the candidates were similar between the PMP cell lines, but within the colon cancer cell panel, there was considerable variation of the expression of these candidate genes, and in turn, several differed to the PMP lines—as illustrated in Figure 5. These experiments also confirmed that gene expression of ARHGAP24 is highly elevated among the PMP lines compared with the 5 colon cancer cell lines.

### Gene profiles differ in PMP compared with colorectal neoplasia

**Figure 6:**
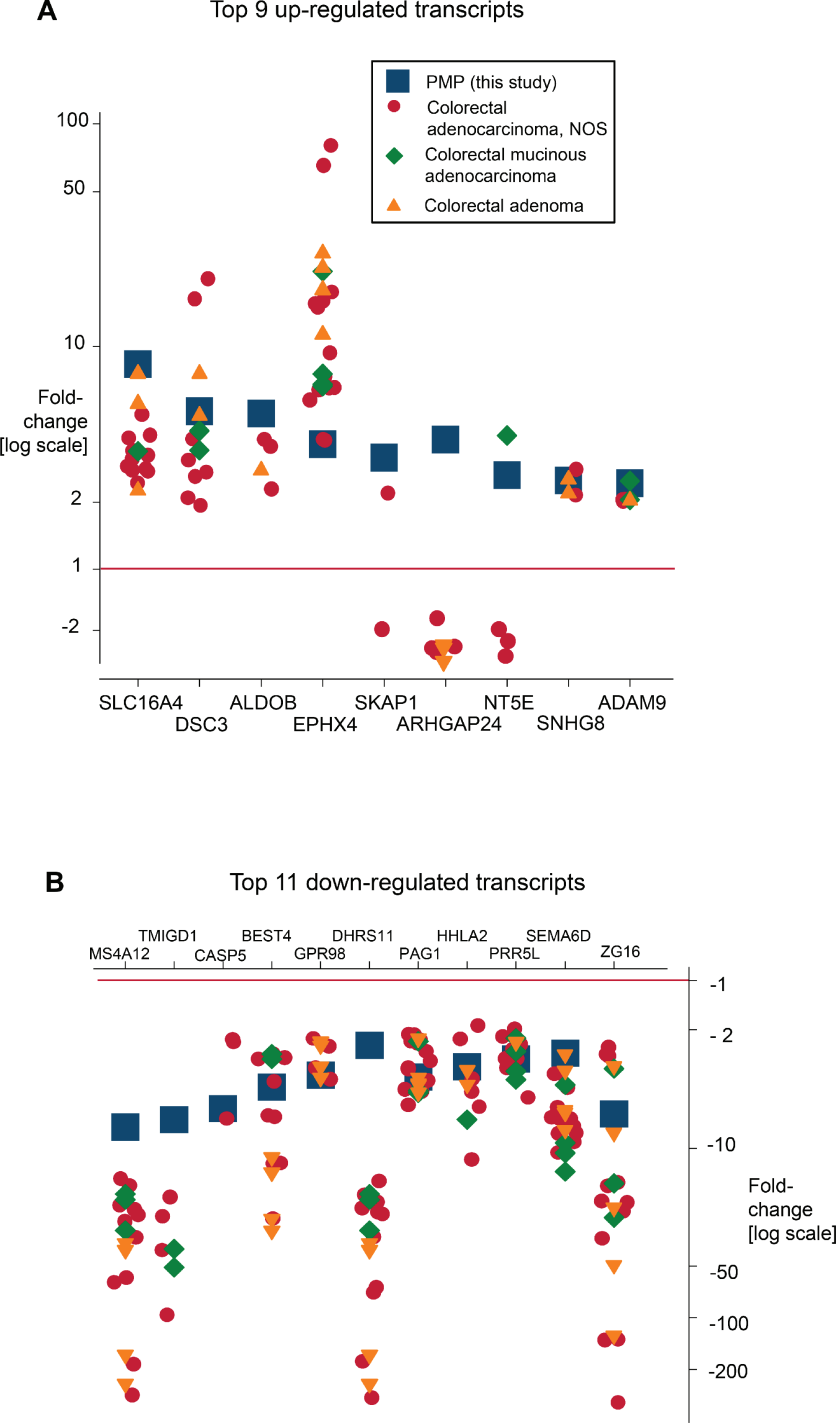
The transcription profile of PMP was compared with those of colorectal tumours (colorectal adenocarcinoma, mucinous adenocarcinoma and adenoma) by comparing changes in transcript level in the present study with those reported in similar studies in the Oncomine database Changes were reported using similar comparisons (normal vs tumor, mRNA/cDNA input) and similar array platforms were recorded. The fold change observed for each transcript was then plotted for each comparison on the log scale. For PMP, **(A)** nine up-regulated transcripts (SLC16A4, DSC3, ALDOB, EPHX4, SKAP1, ARHGAP24, NTE5, SNHG8, ADAM9), and **(B)** eleven down-regulated transcripts (MS4A12, TMIGD1, CASP5, BEST4, GPR98, DHRS11, PAG1, HHLA2, PRR5L, SEMA6D, ZG16) were compared with the same gene expressions in colorectal tumors. Clear patterns emerged (summarized in main manuscript).

The transcription profiles of PMP were compared with those of colorectal neoplasia (colorectal adenocarcinoma, mucinous adenocarcinoma and adenoma) by comparing changes in transcript level in this study with those reported in equivalent studies in the Oncomine database. The fold change observed for each transcript was then plotted for each comparison on the log scale. We compared the top nine up-regulated transcripts (SLC16A4, DSC3, ALDOB, EPHX4, SKAP1, ARHGAP24, NTE5, SNHG8, ADAM9) and top eleven down-regulated transcripts (MS4A12, TMIGD1, CASP5, BEST4, GPR98, DHRS11, PAG1, HHLA2, PRR5L, SEMA6D, ZG16) from our PMP gene list, which were also present within the Oncomine database. Several transcripts were up-regulated in PMP and in colorectal tumors, but the extent of the changes were distinct. Thus, up-regulated expression was greater in PMP for SLC16A4, DSC3, ALDOB compared with colorectal tumors. However, three transcripts (SKAP1, ARHGAP24, and NT5E) were up-regulated in PMP but generally down-regulated in colorectal tumors (Figure 6A). For down-regulated transcripts, there was commonality with colorectal tumors, although the extent of reduced expression was generally greater in colorectal tumors compared with PMP (Figure 6B).

### Preliminary cytotoxicity testing

**Figure 7:**
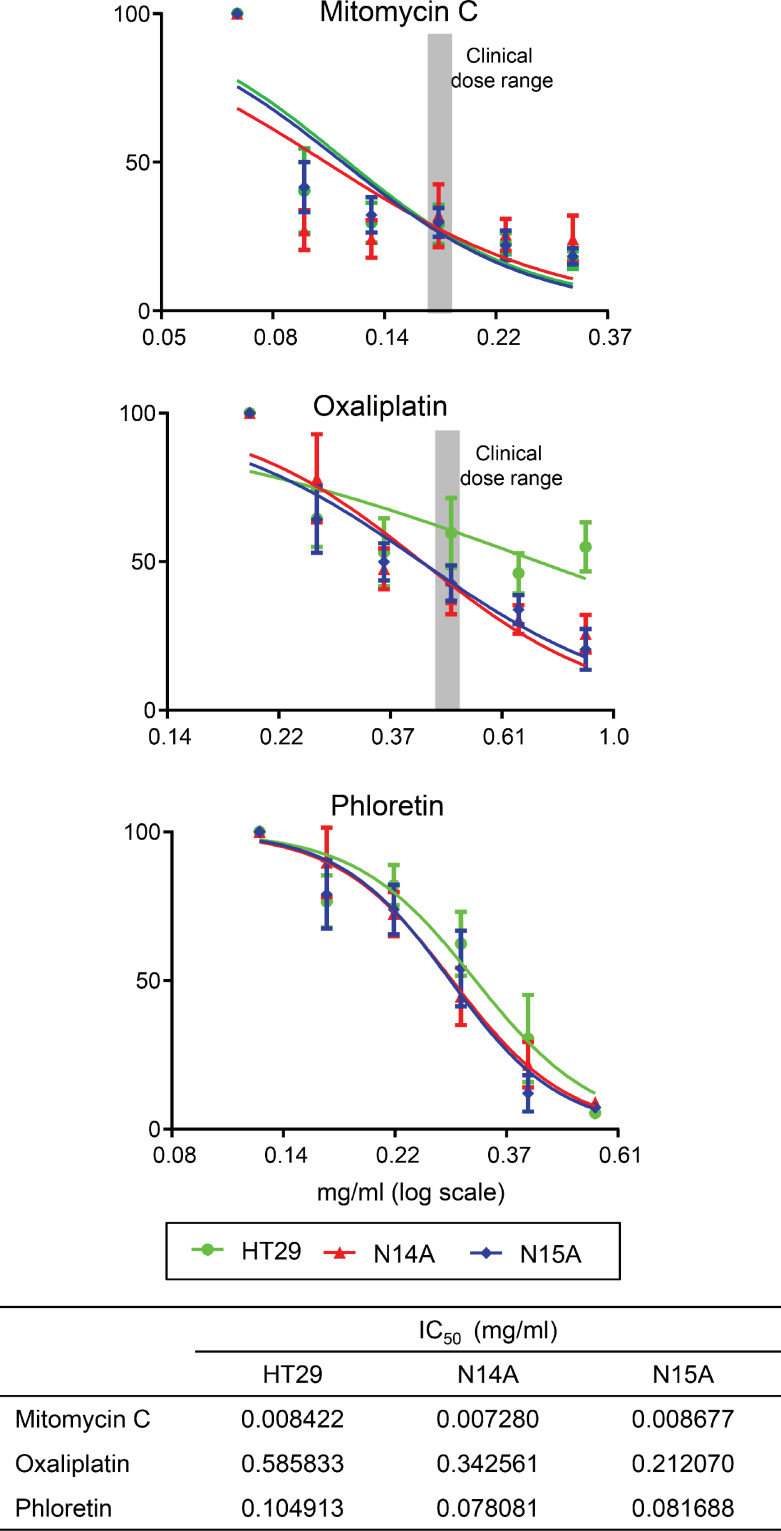
We tested the *in vitro* sensitivity of the immortalized cell lines (N14A, N15A) and a comparator colon cancer cell line, HT-29, against commonly used chemotherapy agents—mitomycin C and oxaliplatin—and Phloretin, an apple metabolite previously reported as an inhibitor of lactate and pyruvate transport, and used here as an example of a pathway inhibitor To determine IC_50_ values, cells were seeded in complete medium and allowed to adhere for 24 h prior to drug treatment. Media were replaced following 24 h exposure to the drugs and the cells were incubated under normal culture conditions for 4 days before the MMT assay. For mitomycin and oxaliplatin, we selected a range of concentrations centred round clinically relevant values (grey-shaded area). The cytotoxic efficacy was evaluated after determining respective IC_50_ values, the concentrations that reduced growth by 50%, using GraphPad Prism software (bottom panel). All experiments were performed at 37°C. Data in the graphs represent the mean ± SD of at least three independent experiments performed in triplicate.

To test the utility of the cell lines, N14A and N15A, as pre-clinical models of PMP, the sensitivity of these cells to Mitomycin C (MMC) and Oxaliplatin, agents currently used in the treatment of PMP, were determined (Figure 7). The dose response curves (using doses spanning the typical clinical maximum dose range) and IC_50_ values indicate that the two PMP cells were more sensitive to MMC than Oxaliplatin, and that the sensitivities were similar for MMC and slightly less for Oxaliplatin, compared with the same agents against the colon cell line, HT-29. As a proof of principle that these cells might be used as a pre-clinical tool for novel agents, we tested the sensitivity of the PMP cell to phloretin, an apple metabolite previously reported as an inhibitor of lactate and pyruvate transport [18] and used to inhibit monocarboxylate transporters (similar to SLC16A4) [19]. We found IC_50_ values equivalent to those for Oxaliplatin, and again, the sensitivities were very similar to those against the colon cell line, HT-29.

## DISCUSSION

### Main findings

We have undertaken a global gene exon array analysis in histologically confirmed PMP tissue, and identified differentially genes as candidates that may underpin the unique natural history of this malignancy, and shown that these dysregulated genes and their protein products are representative in a series of human PMP samples. For the first time, we additionally developed two immortalized PMP cell lines, N14A and N15A, and established a platform for future pre-clinical anti-tumor drug testing and oncogene discovery in PMP. We cross-checked these novel cell lines against the differentially expressed genes (from the array analyses) using qPCR and found consistencies. Finally, we demonstrated that the gene profiles in PMP are distinct from those of colorectal tumor libraries and commonly used colon cell lines.

### Context of literature

The basic pathobiology of PMP is understudied, reflecting its rarity and its historic pathological confusion with other neoplastic processes, namely ovarian cancer. Previous studies have reported on altered expression of selected protein pathways [7–10] and selected genetic mutations [11–14]. One recent study [20] reported a genomic profiling analysis in 19 PMP specimens using next-generation sequencing, but this was limited to a 48 cancer-related gene amplicon. By contrast, this current study performed a global expression analysis and identified a number of potential candidate genes. First, SLC16A4, a monocarboxylate transporter (of lactate, pyruvate and other monocarboxylates), was the most significantly overexpressed gene in our analysis. SLC16A4 is expressed in the intestine [21, 22] and is a potential therapeutic target in conditions characterized by hypoxia [23]. Whilst we speculate that hypoxia is not a dominant characteristic of PMP, glucose and fatty acid metabolism of PMP cells might be dysregulated, and may in part explain the production of the large amount of mucus observed in this disease. Highly elevated SLC16A4 expression appears to be PMP specific, with equivalent elevated levels limited to only some colorectal tumors and cancer cell lines. The second overexpressed candidate gene was desmocollin-3 (DSC3), a member of the cadherin family expressed in epithelial cells [24]. Switching from desmocollin-2 to desmocillin-3 has been noted in colorectal cancer development [25] and the methylation of the DSC3 promoter is a prognostic marker of colorectal cancer [26]. PMP is unique in its pattern of growth and dissemination as it exists as strings of cells within pools of mucus loosely attached to surrounding tissue. Overexpression of DSC3 might maintain the cell-to-cell contacts leading to the string-like morphology. Third was Aldolase B (ALDOB), an enzyme involved in fructose metabolism, which is known to be overexpressed in ulcerative colitis [27]. Loss of ALDOB is associated with early recurrence and poor prognosis in hepatocellular carcinoma [28]. The up-regulated expression of ALDOB in PMP may reflect the potential high metabolic requirements to sustain the production of large amounts of mucin. Fourth was epoxide hydrolase 4 (EPHX4), an enzyme involved in carcinogen inactivation with reduced levels of epoxide hydrolase activity and linked to increased colorectal cancer risk [29]. Elevated levels of EPHX4 in PMP may reflect its relatively differentiated state.

Of the down-regulated gene, MS4A12 is a colon selective calcium channel with expression normally restricted to the apical membrane of colonocytes. Loss of MS4A12 in Lovo cells attenuates EGFR mediated effects, resulting in reduced invasion, chemotactic motility and proliferation [17], which matches with the known phenotype of PMP (low mitogenic potential and no distant metastasis formed) [7]. MS4A12 is elevated in colorectal cancer, but down-regulated in our PMP samples despite being a known CDX2 transcriptional target, often up-regulated in PMP [30]. This may reflect different pathways initiated by CDX2 in appendiceal tissue in comparison with colonic tissue. TMIGD1, (transmembrane and immunoglobulin domain containing protein-1) is a relatively unknown protein, though with known expression in the colon (Unigene database). Caspase-5 is involved in the inflammatory response and frameshifts are often found in endometrial [31] and lung [32] cancers. In addition, caspase-5 cleaves Max, part of the Myc network of transcription factors, and therefore, may play a role in tumorigenesis [33].

### Limitations and strengths

There are potential limitations to this study. First, PMP is a heterogeneous clinico-pathological disease, such that our modest sample size may not have captured its diversity. Nonetheless, the homogeneity of the gene microarray analysis was noteworthy and allowed us to derive and generate hypotheses based on small sample numbers. Second, we did not measure mRNA expression, other than for SLC16A4. However, previous studies have noted strong correlations between protein and mRNA expression in colorectal tissue [34], and we propose a similar association in PMP tissue. Third, we utilized normal colonic epithelia in our micro-array analyses which may differ in its gene expression patterns to normal appendiceal epithelia. However, we confirmed similarities in the expression of the protein products of our candidate genes in both colonic and appendiceal tissue. Finally, a potential limitation was the use of the SV40 large T antigen to immortalize the cell lines, which may modify the genetic characteristics of these cells. Nonetheless, this technology is commonly used in other cell line systems, and for our PMP lines, the expressions of the key candidate genes mirrored those from the *in vivo* human PMP samples.

Strengths of the study are the following. First, working within the framework of national treatment center, we optimized protocols for the collection of fresh ‘disease-rich’ tissue and pathological confirmation. Second, as part of our previous published work [7], a learning set of PMP samples was used to explore histopathological features for IHC. This is critical, as the cellular components on PMP are sparse and many sections are required to establish optimal representation. Third, previous attempts to (spontaneously) develop a PMP cell line have resulted in limited success [7]; we have improved on this with the immortalization of the cells using a lentivirus to express the entire SV40 genome; successfully used in other intestinal *in vitro* systems, and here, achieving viability to over 20 passages. Fourth, to validate our exon array findings, we tested gene and protein product expression in a panel of representative PMP samples. Finally, we demonstrated that the genetic make-up of PMP is distinct from that for colorectal adenoma, colorectal adenocarcinoma, and a number of colon cancer cell line.

### Clinical implications and future research

For patients with PMP, there are broadly two clinical scenarios in which chemotherapy may have a role: in patients with advanced unresectable disease, and as adjuvant to cytoreductive surgery, typically administered as HIPEC, at the time of surgery. For the former, clinical series demonstrate poor survival (median survival ranges from 12 to 18 months) after debulking surgery [4, 5, 15], reflecting poor response to systemic chemotherapy. We previously reported a phase II trial in this patient group with only modest benefit [16]. Thus, there is a clear clinical need to develop and test systemic agents with proven pre-clinical efficacy. But until now, we had no pre-clinical *in vitro* models for such testing.

For the clinical scenario of intra-operative chemotherapy perfusion, there is considerable discussion around maximizing agent delivery (within the abdominal cavity), while minimizing systemic absorption [35, 36]. Yet, there is a lack of data on the cytotoxic characteristics of the chemotherapy agents used. We can now address this using these pre-clinical *in vitro* models.

The establishment of two immortalized PMP lines provides a platform to undertake pre-clinical testing at several levels. First, one can optimize existing clinically-used protocols, and for example, expand the experiments with MMC and oxaliplatin reported in this study. Second, one may test for novel anti-PMP agents, either as selected mechanism-driven approaches, such as the phloretin experiment, or undertake high-throughput screening for multiple agents. Third, as other investigators develop new agents for mucinous subtypes of breast, colorectal and ovarian cancers, these can be rapidly tested in the PMP cell lines. Fourth, these cells will offer a sustainable source for animal xenograft studies, which until now have relied upon primary cells for each experiment [37]. Finally, this platform will allow the expansion of mechanistic *in vitro* studies identifying the key molecular pathology underpinning the initiation and development of PMP.

## MATERIALS AND METHODS

### Patients and controls

The primary cell cultures and PMP tissue samples were from histological confirmed cases undergoing cytoreductive surgery at the Manchester Peritoneal Tumour Service, The Christie NHS Foundation Trust, United Kingdom, a national center for the treatment of tumors of appendiceal origin [5]. Comparative tissues were: normal colonic (*n* = 8) and appendiceal (*n* = 2) mucosa versus neoplastic appendiceal (*n* = 3) and disseminated (*n* = 7) PMP. Demographic and clinic-pathological characteristics are listed in Supplementary Table S2. Samples were subdivided as: (i) frozen; (ii) formalin-fixed, paraffin embedded tissue blocks; and (iii) taken from the PMP material for primary cell culture. Normal colonic and appendiceal tissues were orientated to produce maximal numbers of longitudinal crypts. Histological identification of PMP areas within specimens was confirmed by staining for cytokeratin 20 (KRT20), MUC2, and CDX2 [10]. All tissue work was performed under local ethics approval (ref 11/NW/0638).

### Gene microarrays

For the gene microarrays, we compared three disseminated plus one appendiceal PMP samples with three samples of normal colonic mucosa. Fresh frozen samples were sectioned and stained with cresyl violet (LCM Staining Kit, Ambion, Life Technologies, Paisley, UK). Epithelial cells were captured from approximately 6 sections per sample using a Leica LMD6000 system, and to avoid any bias in cell lineage full length crypts from normal samples were captured wherever possible. RNA was isolated from each sample immediately after laser capture using a Qiagen RNAeasy plus micro kit (Qiagen, Crawley, West Sussex, UK) as per the manufacturer's instructions. The isolated RNA was quantified using an Agilent bioanalyzer and amplified using a NuGEN WT-Ovation™ RNA Amplification System (NuGEN, Leek, The Netherlands) as recommended by the manufacturer. The amplified RNA was hybridized to Affymetrix Genechip Human Exon 1.0 ST Arrays at the Cancer Research UK Affymetrix Genechip Microarray Service. Probesets were filtered to retain exonic, reliable, uniquely mapping probesets and retained probesets were also filtered according to DABG score (detection above background), to keep only those probesets reliably detected in at least two of the 7 samples.

Principal Components Analysis (PCA) was used to explore overall data structure. Expression data were then summarized to provide a single expression measure per gene, by taking the median of the log_2_ expression of the filtered probesets mapping to a unique gene symbol. PCA was repeated for the gene level expression data and these data were taken forward for further analysis. The R package *limma* was used to identify differentially expressed genes between the diseased versus control groups. Genes were classed as differentially expressed if the log fold change difference between the two groups was greater than 2 and were statistically significant if *p* values were less than 0.05 after adjustment for multiple testing.

Genes were categorized based on key words using Ingenuity Pathway Analysis (IPA^®^) software (Ingenuity Systems, CA, USA http://www.ingenuity.com/products/ipa) and literature searches of the PubMed database using the gene name. The entire differentially regulated gene set was utilized for the analysis (fold change cut-off 2.0), but for presentational purposes, the reported analysis is limited to the top 40 ranked pathways.

### Immunohistochemistry and *in situ* hybridization

All IHC and ISH were performed on 5 μm thick FFPE sections on a BOND-MAX stainer (a standardized automated system) using a Bond Polymer Refine Detection Kit (Leica Microsystems, Milton Keynes, UK). Antigen retrieval was performed using ER1, a pH6.0 HIER solution (Leica Microsystems) for 20 minutes and tissues were stained with antibodies to MUC2 (1/2000, Novacastra, Leica Microsystems), CDX2 (1/2000), Aldolase B (1/500) and Caspase-5 (1/100, Abcam, Cambridge, UK), KRT20 (1/3000) EPHX4 (1/50) and TMIGD1 (1/100, Sigma Aldrich, Poole, UK), MS4A12 (1/1500, Abnova, via Caltag Medsystems, Buckingham, UK), SLC16A4 (1/1500 Novus Biologicals, Cambridge, UK), ARHGAP24 (1/1000, Proteintech, Manchester, UK) and DSC3 (1/100, Acris via 2BScientific, Upper Heyford, UK) using IHC BOND-MAX protocol F, and appropriate positive and negative controls. Slides were scanned using a Leica SCN400 slide scanner. Images were analyzed using Definiens Tissue Studio 2.0 software. Briefly, the software was trained using composer to identify the epithelial component of the tissue and then to quantify the staining intensity within this component. All staining intensities reported are a mean whole cell intensity of those cells within the epithelial component of the entire tissue section. Software algorithms are available upon request.

ISH probes were oligonucleotides from Invitrogen (Paisley, UK) against SLC16A4 (5′-Flourescein-TAAGGGCTCCTGTCCAGTCATACA-3′) and were designed by the Sigma–Aldrich probe design service. Positive and negative control probes were purchased from Leica Microsystems. Probes were detected using antibodies against Fluorescein (Leica Microsystems) or Biotin (Life Technologies) using an optimized ISH protocol on a BOND-MAX autostainer.

### Cell culture and characterization

Cell lines were cultured in a humidified environment containing 5% CO_2_ and were regularly monitored for mycoplasma infection, and found to be negative. PMP cell lines were isolated as follows. Surgical samples were collected and transported on ice in PBS containing 10 units Penicillin/0.1 mg Streptomycin per ml (Sigma Aldrich) and 0.25 μl/ml Amphotericin B (Life Technologies). Tissue was dissected macroscopically to remove excess mucous and normal tissue and then minced using two scalpels. The resulting tissue was cultured in high-glucose Dulbecco's modified Eagle's medium (DMEM) (Sigma Aldrich) supplemented with 10% FCS, 25 mM HEPES, 5 μg/ml Insulin, 10 mM L-Glutamine, 10 units Penicillin/0.1 mg Streptomycin (all Sigma Aldrich) and 0.25 μl/ml Amphotericin B (Life Technologies). After 1 to 5 days (depending upon the amount and type of tissue) tissue fragments and debris was removed and fresh media added. Cells were harvested 24 h later by chelation using PBS containing 3 mM EDTA and 0.6 mM DTT to selectively detach epithelial cells. A proportion of the cells were plated into 6 well plates and the remainder cryogenically stored in FCS containing 10% DMSO. The cells remaining in culture were then infected with Lenti-SV40 lentivirus (NBS Biologicals, Huntingdon, UK) at an MOI > 10 as per the manufacturers' instructions. Media was replaced after 16 h and then cells passaged three days later. After two passages a sample of the cells was cryogenically stored and the remainder plated at low density to allow clonal expansion. Individual colonies which exhibited epithelial morphology were then selected and expanded further for characterization.

Cell lines were validated by STR analysis by the Molecular Biology Core facility at the Paterson Institute for Cancer Research, Manchester, United Kingdom, using the Powerplex 21 system (Promega, Southampton, UK) and compared with the ATCC database and genomic DNA isolated from FFPE patient samples using a QIAamp DNA FFPE Tissue kit (Qiagen). Cell lines were tested for the presence of the PMP markers by immunoblotting using antibodies to CDX2 (Abcam, Cambridge, UK) and KRT20 (Sigma Aldrich). For additional characterization using qPCR RNA was isolated using a Qiagen RNeasey plus micro kit and reverse transcribed using a High Capacity RNA-to-cDNA kit (Life Technologies). The resulting cDNA was then used in Taqman gene expression assays for Beta-Actin (Hs99999903_m1) and SLC16A4 (Hs01006127_m1), using Taqman Universal PCR Master Mix on an ABI 7900HT Q-PCR machine.

For comparative experiments, five colon cancer cell lines were used: HCT 116, Caco2, HT-29, Lovo (all from American Type Culture Collection, ATCC) and C32 (C32 (a gift from Professor Mohammod Ilyas, Nottingham, UK) and maintained as previously described [38].

### Oncomine data

The transcription profiles for PMP were compared with those for colorectal tumors by comparing changes in transcript levels in this study with those reported in studies reporting similar methodologies and platforms (normal vs tumor, mRNA/cDNA input) on colorectal neoplasia (adenomas, adenocarcinoma, and mucinous adenocarcinoma) identified in the Oncomine database (https://www.oncomine.org/resource/login.html). The fold change (up- and down-regulated) observed for each transcript was then plotted for each comparison.

### Cytotoxicity testing

Cells were plated in 96 well plates and allowed to reach confluency (approximately 1 day) to enter the plateau phase of growth, mimicking the non-proliferating state of PMP *in vivo*, and the media replaced. Cells were then exposed to a dose range of Mitomycin C, Oxaliplatin (R&D Systems, Abingdon, UK), or Phloretin (Sigma Aldrich) for 60 minutes at 37°C. Media was then replaced and the cells cultured under normal conditions for a further 4 days. Cellular metabolism was assessed using the MTT assay by addition of 1/10 volume (10 μl) of 5 mg/ml Thiazolyl Blue Tetrazolium Bromide (Sigma Aldrich) and incubation for 4 h. 75 μl of media was then removed and 50 μl DMSO added to solubilize the MTT crystals. Plates were incubated at 37°C for 10 minutes and then absorbance measured at 540 nm. Growth curves were fitted using a four parameter sigmoidal algorithm in GraphPad Prism 6 (GraphPad Software Inc. La Jolla, CA, USA) and IC_50_ values derived.

### Statistical analysis

Comparisons of continuous variables were performed using Student's *t*-test (Stata™ 11.1, College Station, TX, USA).

## SUPPLEMENTARY FIGURE AND TABLES


