# Supplementary material for: Global gene expression in pseudomyxoma peritonei, with parallel development of two immortalized cell lines
Source: Oncotarget. 2015 Apr 13;6(13):10786–800. doi: 10.18632/oncotarget.3198 (PMC4484419; doi:10.18632/oncotarget.3198)
Supplement: Supplementary file 1 [file oncotarget-06-10786-s001.pdf]

SUPPLEMENTARY FIGURE AND TABLES

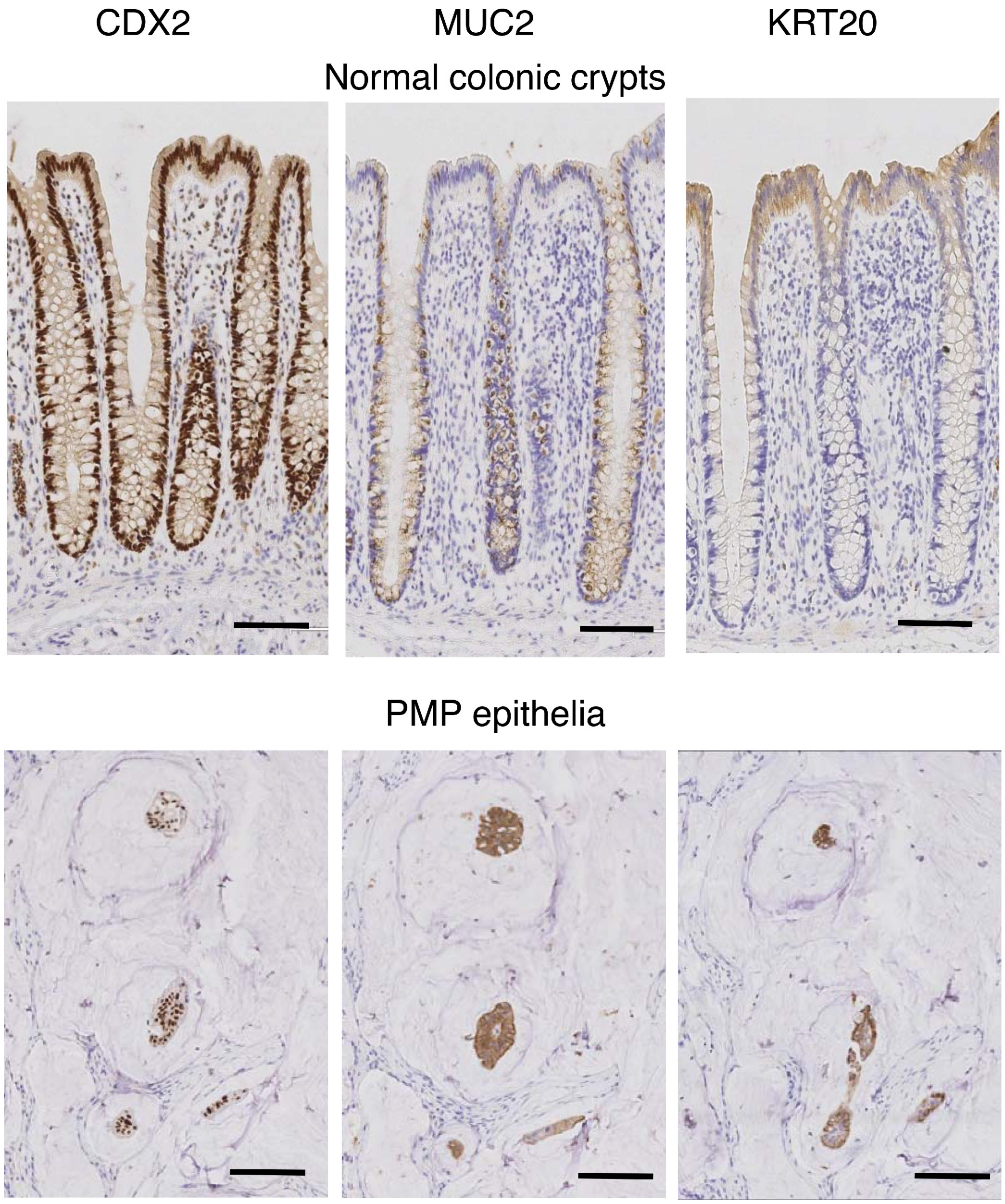

Supplementary Figure S1: Immunohistochemical staining showed no difference in the intensity of stain for the clinically used tissue markers, CDX2 and KRT20. This reflects the biology of these markers. CDX2 is commonly used as a marker of differentiation in colorectal cancer[1] and was found to be distributed throughout crypts in the normal tissue and was present in the majority of the PMP cells. Expression levels of CDX2 did vary within the PMP tissue which may reflect a partially de-differentiated state in some of the cells. KRT20 (Cytokeratin 20) is another differentiation marker and its expression is limited to the colonic table in the normal tissue whereas in PMP it was found to be expressed ubiquitously. We additionally stained for MUC2: its expression was limited to the goblet cells within the normal tissue, whereas it was present in all PMP cells and the intracellular distribution was diffuse within the PMP tissue whereas it is granular in normal tissue.

1. Guo R-J, Suh ER, Lynch JP. The role of Cdx proteins in intestinal development and cancer. Cancer Biology & Therapy. 2004; 3:593–601.

Supplementary Table S1: Genes differentially expressed between PMP and normal colonic mucosa (genes highlighted in red were considered as candidate genes and studied further)

GENE SYMBOL	Description	Log-fold Change	*p* value
**Up-regulated in PMP**			
**SLC16A4***	Proton-linked monocarboxylate transporter.	8.373903	0.00611
**DSC3***	Component of intercellular desmosome junctions.	5.142457	0.011396
SNAPC5	Part of the SNAPc complex required for the transcription of both RNA polymerase II and III small-nuclear RNA genes.	4.429103	0.014065
RP11-1143G9.4	Antisense to Lysozyme	3.639942	0.018252
CTD-2589M5.4	Novel Antisense	5.75735	0.021407
**ALDOB***	Adolase. Fructose-1,6-bisphosphate aldolase (EC 4.1.2.13) is a tetrameric glycolytic enzyme that catalyzes the reversible conversion of fructose-1,6-bisphosphate to glyceraldehyde 3-phosphate and dihydroxyacetone phosphate.	5.012758	0.021407
**EPHX4***	Hydrolase activity	3.651662	0.021407
SKAP1	Positively regulates T-cell receptor signaling by enhancing the MAP kinase pathway.	3.16588	0.021407
SLC6A14	Mediates the uptake of a broad range of neutral and cationic amino acids (with the exception of proline) in a Na+/Cl--dependent manner.	6.107397	0.022284
C15orf41	Uncharacterised	3.197417	0.022284
SLC5A1	Belongs to the sodium:solute symporter (SSF) (TC 2.A.21) family	3.09706	0.022284
PTPN14	cytoskeleton - dephosphorylates beta catenin, regulates TGF-beta signalling	2.589703	0.022284
FAM57A	amino acid transport and glutathione metabolism	3.229197	0.027531
LYZ	primarily a bacteriolytic function	4.178017	0.028317

(*Continued*)

GENE SYMBOL	Description	Log-fold Change	*p* value
C11orf1	Nuclear - unknown function	3.01615	0.028418
**ARHGAP24**	Rho GTPase-activating protein involved in cell polarity, cell morphology and cytoskeletal organization.	3.829627	0.028434
BTRC	Substrate recognition component of a SCF (SKP1-CUL1-F-box protein) E3 ubiquitin-protein ligase complex - mediates the ubiquitination of CTNNB1 and participates in Wnt signaling.	2.590747	0.028434
NT5E	Involved in nucleotide catabolic process	2.635723	0.03625
SNHG8	small nucleolar RNA host gene 8	2.493767	0.03625
ADAM9	Probable zinc protease. May mediate cell-cell or cell-matrix interactions.	2.431713	0.03625
ALOX5	Arachidonate 5-lipoxygenase	2.718643	0.037504
PDE10A	Plays a role in signal transduction by regulating the intracellular concentration of cyclic nucleotides.	3.07907	0.038132
RASA3	Inhibitory regulator of the Ras-cyclic AMP pathway.	2.109607	0.039276
ARL3	Small GTP-binding protein - required for normal cytokinesis and cilia signaling.	2.537887	0.039383
GABRP	pI subunit of GABA(A) receptor	3.3324	0.040541
PTPN13	peptidyl-tyrosine dephosphorylation, AKA Fas-associated phosphatase-1	2.943257	0.04238
KIAA0895	Unknown	3.162537	0.043404
**Down-regulated in PMP**			
**MS4A12***	May be involved in signal transduction as a component of a multimeric receptor complex.	−7.30833	0.00611
**TMIGD1***	Transmembrane and immunoglobulin domain-containing protein 1	−6.61371	0.00611

(*Continued*)

GENE SYMBOL	Description	Log-fold Change	*p* value
BEST4	Forms calcium-sensitive chloride channels. Permeable to bicarbonate.	−4.24565	0.008264
AL928768.3	Novel lincRNA	−6.37649	0.011867
IGKJ5	immunoglobulin kappa joining 5	−6.07338	0.014065
CDKN2B-AS1	Known antisense to Cyclin-dependent kinase inhibitor 2A	−5.09636	0.014065
IGHV3-23	immunoglobulin heavy variable 3-23	−3.73304	0.021407
**CASP5***	Mediator of programmed cell death (apoptosis).	−5.71187	0.022284
GPR98	Receptor that may have an important role in the development of the central nervous system.	−3.6213	0.022284
RP3-405J10.4	Novel antisense to LIMA1 (LIM domain and actin-binding protein 1) which is down regulated in some cancer cell lines and Binds to actin monomers and filaments.	−3.01579	0.022284
AC013553.1	Novel lincRNA	−2.80339	0.022284
DHRS11	Dehydrogenase/reductase SDR family member 11	−2.40573	0.025247
IBTK	Acts as an inhibitor of BTK tyrosine kinase activity	−4.04263	0.027531
**PAG1**	Negatively regulates TCR (T-cell antigen receptor)-mediated signaling in T-cells and FCER1 (high affinity immunoglobulin epsilon receptor)-mediated signaling in mast cells.	−3.70044	0.02788
CTB-118N6.3	Novel antisense to SEMA6A (Semaphorin-6A - Cell surface receptor for PLXNA2 that plays an important role in cell-cell signaling.)	−4.15585	0.028418
HHLA2	Human endogenous retrovirus-H long terminal repeat-associating protein 2	−3.23025	0.028418

(*Continued*)

GENE SYMBOL	Description	Log-fold Change	*p* value
PRR5L	May be part of the TORC2 complex which plays a critical role in AKT1 'Ser-473' phosphorylation	−2.89777	0.028418
SEMA6D	Shows growth cone collapsing activity on dorsal root ganglion (DRG) neurons *in vitro*.	−2.68542	0.028418
ZG16	May play a role in protein trafficking.	−6.12767	0.033641
TBC1D4-AS1	Putative antisense of TBC1D4 (AS160 - an AKT substrate.)	−5.96682	0.033641
ATAD5	Involved in DNA damage response.	−2.54	0.033641
ACE2	Carboxypeptidase which converts angiotensin I to angiotensin 1-9, a peptide of unknown function, and angiotensin II to angiotensin 1-7, a vasodilator.	−3.89078	0.03625
AC130454.2	Genomic region encoding Protein kinase C beta type (Calcium-activated, phospholipid- and diacylglycerol (DAG)-dependent serine/threonine-protein kinase	−3.02803	0.03625
TRPM6	Essential ion channel and serine/threonine-protein kinase. Has an important role in epithelial magnesium transport and in the active magnesium absorption in the gut and kidney.	−3.82004	0.036358
IGJ	Immunoglobulin J chain serves to link two monomer units of either IgM or IgA.	−6.29508	0.037504
ARHGAP17	Rho GTPase-activating protein involved in the maintenance of tight junction by regulating the activity of CDC42	−2.33299	0.038132
PRKAR2B	Regulatory subunit of the cAMP-dependent protein kinases involved in cAMP signaling in cells.	−3.8791	0.04238

(*Continued*)

GENE SYMBOL	Description	Log-fold Change	*p* value
TEX11	Testis-expressed sequence 11 protein	−2.476	0.04238
RFWD2	E3 ubiquitin-protein ligase that mediates ubiquitination and subsequent proteasomal degradation of target proteins.	−2.2422	0.04238
C7orf31	Uncharacterized protein C7orf31	−2.47692	0.042567
REV3L	DNA polymerase zeta catalytic subunit	−4.21409	0.04349
FAM55A	NXPE family member 1 (secreted)	−4.14822	0.044994
AC069513.3	Novel processed transcript within the MUC20 gene	−3.98019	0.049298
D87024.2	Known miRNA	−4.39847	0.049919

*Specifically studied in the ISH and IHC validation experiments.

Supplementary Table S2: Patient demographics and clinic-pathological characteristics

	Normal colon	Normal appendix	Appendiceal PMP	Disseminated PMP
Patients	8*	2*	4*	7*
Median age (range), years	51 (33–76)	44 (37–51)	49 (29–73)	57 (42–65)
Men: women	3:5	1:1	0:4	3:4
PMP Classification				
DPAM	N/A	N/A	2	5
PMCA-I	N/A	N/A	2	2

*Numbers reflect the total number of human samples used in the study, and may not equal the numbers in the IHC experiments (Figure 3).
